# Three-Dimensional Bi_2_Te_3_ Networks of Interconnected Nanowires: Synthesis and Optimization

**DOI:** 10.3390/nano8050345

**Published:** 2018-05-18

**Authors:** Alejandra Ruiz-Clavijo, Olga Caballero-Calero, Marisol Martín-González

**Affiliations:** Instituto de Micro y Nanotecnología, IMN-CNM, CSIC (CEI UAM+CSIC) Isaac Newton, 8, E-28760 Madrid, Spain; alejandra.ruizclavijo.garcia-serrano@csic.es (A.R.-C.); olga.caballero@csic.es (O.C.-C.)

**Keywords:** electrochemistry, bismuth telluride, nanowires, 3D–AAO, nanonetwork, metamaterial

## Abstract

Self-standing Bi_2_Te_3_ networks of interconnected nanowires were fabricated in three-dimensional porous anodic alumina templates (3D–AAO) with a porous structure spreading in all three spatial dimensions. Pulsed electrodeposition parameters were optimized to grow highly oriented Bi_2_Te_3_ interconnected nanowires with stoichiometric composition inside those 3D–AAO templates. The nanowire networks were analyzed by X-ray diffraction (XRD), scanning electron microscopy (SEM), energy dispersive X-ray analysis (EDX), and Raman spectroscopy. The results are compared to those obtained in films and 1D nanowires grown under similar conditions. The crystalline structure and composition of the 3D Bi–Te nanowire network are finely tuned by controlling the applied voltage and the relaxation time off at zero current density during the deposition. With this fabrication method, and controlling the electrodeposition parameters, stoichiometric Bi_2_Te_3_ networks of interconnected nanowires have been obtained, with a preferential orientation along [1 1 0], which makes them optimal candidates for out-of-plane thermoelectric applications. Moreover, the templates in which they are grown can be dissolved and the network of interconnected nanowires is self-standing without affecting its composition and orientation properties.

## 1. Introduction

Thermoelectric devices have raised the interest of the scientific community due to their capability to convert thermal energy directly into electrical energy and vice versa, as in power generators and coolers. The necessity of developing alternative energy sources other than those based on fossils fuels has brought thermoelectric materials into the spotlight. However, the applications of thermoelectric based devices are still limited as consequence of their low efficiency. The performance of a thermoelectric device is proportional to its figure of merit, *ZT*, which is a dimensionless quantity defined as $ZT=\sigma \cdot S^{2}\cdot T/\kappa$, where *T*, *S*, *σ*, and *κ* are the absolute temperature, Seebeck coefficient, electrical conductivity, and thermal conductivity, respectively. The ideal thermoelectric material should have a high Seebeck coefficient, high electrical conductivity, and low thermal conductivity; these requirements are not easily met, though [1].

A bismuth telluride semiconductor is one of the most commonly used materials for thermoelectric applications near room temperature. Further enhancement of its thermoelectric performance can be achieved by nanostructuration, more specifically by reducing its dimensionality, and so increasing the surface to volume ratio. One way of doing that is by fabricating nanowires. There has been reported a reduction of the thermal conductivity, *κ*, of electrodeposited Bi_2_Te_3_ nanowires when decreasing their diameter from 200 to 20 nm [2], as a consequence of the increase of the acoustic phonon scattering at the interfaces due to a higher surface to volume ratio. 

However, the implementation of these nanostructures into actual devices is not straightforward. On the one hand, these nanowires are electrochemically grown inside a template, being Anodic Aluminum Oxide (AAO) [3,4] membranes the most used. Nevertheless, AAOs can diminish the thermoelectric performance of the whole composite, given that their thermal conductivity can be even higher than that of the nanowires embedded inside. On the other hand, if one thinks of dissolving the AAO matrix, the resulting nanowires would collapse, given that they have no mechanical support to hold them. Another issue to take into account is the difficulty to measure the transport properties at the nanoscale, in order to characterize the produced nanowires [5,6]. 

Nowadays there is much research devoted to different ways of nano-structuring, not only in the way of nanowires, but in order to attain different morphologies at the nanoscale for a variety of applications, such as the recent development of high performance Li/S batteries encapsulated in graphene nanosheets [7], nanofiber membranes used for desalinization of seawater [8], nanotubes used as functionalized nanocavities for drug delivery [9], or nanospheres to study the morphology of porous structures and enhance mass transport [10]. In this line of creating new structures at the nanoscale, we have recently developed novel AAO templates that we call three-dimensional AAO templates (3D–AAO). The fabrication involves a two-step anodization process: a first 24 h anodization at constant potential in sulfuric acid and a second anodization during which a series of different voltages, corresponding to mild and hard anodization, are applied, as reported in a previous work [11]. The final 3D–AAO structures consist of longitudinal pores with a highly ordered hexagonal arrangement that is interconnected to its nearest six pore neighbors by transversal nanochannels. It is worth noting that these 3D–AAO structures can also be made in non-flat surfaces such as wires [12].

In this paper, we report an in-depth characterization and optimization of the growth of self-standing 3D Bi_2_Te_3_ nanowire networks by pulsed electrodeposition using these 3D–AAO membranes as templates. And, we will show a comparison of these 3D Bi_2_Te_3_ nanowire networks with the results obtained with the fabrication of 1D nanowire arrays and films. The aim of this work is to study the dependence of the electrodeposition parameters (such as the applied voltage pulses, applied current density, time on and time off pulses, temperature, etc.) with respect to morphology, crystalline orientation, and stoichiometry of the 3D Bi_2_Te_3_ nanowire network structure. The final goals are determining the optimum fabrication conditions and seeing the differences when compared with normal 1D nanowire arrays or films. It is worth mentioning that these 3D Bi_2_Te_3_ nanowire networks are self-standing, mechanically stable, and easy to manipulate with a pair of tweezers once the 3D–AAO membrane is selectively removed. So, they could be used in the fabrication of a thermoelectric device based on Bi_2_Te_3_ nanowire arrays in the future.

## 2. Materials and Methods 

The 3D–AAO template was fabricated by a two-step anodization process in 0.3 M sulfuric acid, with the second step consisting of alternating between mild and hard anodization pulses, as previously described in [11]. After the anodization process, the remaining aluminum substrate was eliminated using HCl/CuCl_2_ solution. A subsequent etching treatment was carried out by 10% H_3_PO_4_ in order to remove the barrier layer, followed by 5% H_3_PO_4_ etching to open up the transversal channels. As a result, a 3D–AAO template consisting of highly ordered nanopores of around 40 nm in diameter, connected with transversal channels of about 20–30 nm in diameter, is obtained. The distance between the transversal channels can be completely tailored by modifying the pulses of the second anodization step.

The electrodeposition process was performed in a conventional three-electrode cell configuration with a Ag/AgCl saturated electrode as the reference, a platinum mesh as counter electrode and the 3D–AAO template with a 5 nm Cr and 150 nm Au layer evaporated in one of its surfaces and then attached to a copper holder with silver paste, acting as working electrode. Also, conventional alumina templates(1D-AAO) of 40 nm diameter longitudinal pores, evaporated with 5 nm Cr and 150 nm Au and attached to a copper holder, were used as a working electrode in order to carry out a comparative study between the growth of 3D nanowire networks and conventional 1D nanowire arrays. The copper holder was then covered with nail polish in order to isolate it electrically from the solution. In the case of the films, a third working electrode, consisting of a silicon substrate covered with 5 nm Cr and 150 nm Au was used for the comparison. The electrolyte solution used was the same in all the cases and consists of 0.90 × 10^−2^ M Bi^3+^, 1 × 10^−2^ M HTeO_2_^+^, and 1 M HNO_3_. The solution was prepared from Aldrich^(R)^ Bismuth pieces (99.999%), Aldrich^(R)^ Tellurium powder (99.997%), and Panreac 65% nitric acid. Nitric acid is used because H^+^ acts as the supporting electrolyte of the solution and NO_3_^−^ as counterion. The preparation of the solution required several steps: Initially, Bi^3+^ was dissolved in nitric acid. Then the Tellurium powder and de-ionized water are added to the solution, in that order.

Bi_2_Te_3_ was grown by pulsed electrodeposition, switching between a potenciostatic and galvanostatic mode. The applied pulses were alternated between a certain constant potential, during a time on of 1 s and zero current density, at different time off, as reported in previous works [13,14]. The applied potential versus Ag/AgCl in the potentiostatic part of the pulses was determined by a cyclic voltammetric (CV) curve performed prior to each deposition. A bi-potentiostat (Autolab PGSTAT 302) was used with the customized software Nova 1.10 to obtain the cyclic voltammograms and to control the potenciostatic/galvanostatic pulses. Once the deposit was performed, the 3D–AAO matrix or the 1D-AAO matrix (where 1D-AAO stands for conventional anodic alumina with similar diameter pore than the 3D but without transversal interconnecting channels) was detached from the copper holder by immersion in acetone, obtaining a 3D network of Bi_2_Te_3_ nanowires inside the 3D–AAO template or a Bi_2_Te_3_ 1D nanowire array, respectively. In the case of free-standing 3D Bi_2_Te_3_ nanowire networks, the 3D–AAO matrix was then dissolved in 7 wt % H_3_PO_4_ and 1.8 wt % H_2_CrO_4_ for 24 h.

The chemical composition of the different samples were studied by energy dispersive X-ray spectrometry (EDX, Hitachi S-3000 N), where we assume a 5% error in the atomic % value. Raman spectroscopy was used to corroborate EDX results (Jobin-Yvon LabRam HR using a Nb: YAG Laser, *λ* = 532 nm). X-ray diffractometry (XRD, Philips X’Pert PANalytical, with Cu K*α* radiation, *λ* = 0.15418 nm) was employed to assess the preferred crystalline orientation of the formed Bi_2_Te_3_ structures. Structural characterization of both 3D Bi_2_Te_3_ nanowire network embedded inside the 3D–AAO membrane and free-standing wires was carried out by a high-resolution scanning electron microscope (HRSEM, FEI Verios 460) in order to verify that the connecting nanotubes were also filled and that the 3D–AAO structure was effectively replicated. HRSEM cross view measurements were carried out at an accelerating voltage of 2 KV and at a current of 50 pA, while top view images were taken at a lower current of 13 pA, in order to obtain a more detailed image of the surface of the polished sample. The magnification of the images was varied over a range between 15,000–35,000 × and the working distance (WD) used ranged from 3 to 4 mm.

## 3. Results

**Figure 1:**
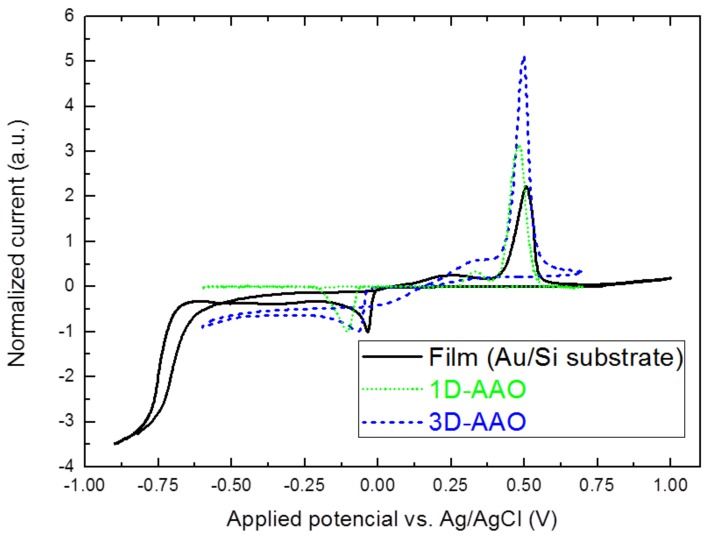
Cyclic voltammetry performed at a scan rate of 10 mV/s, when three different working electrodes are used, namely gold over a silicon substrate (black line), 1D–AAO template (dashed green) and 3D–AAO template (dotted blue).

Firstly, a cyclic voltammetric study was performed to monitor the changes over the reduction peak of Bi^3+^/HTeO_2_^+^ species when growing films, 1D nanowires, and 3D nanowire networks. Cyclic voltammograms were carried out in the above mentioned electrolyte solution, at 0 °C, with a scan rate of 10 mV·s^−1^ over a potential range between −1.0 to 1.0 V applied potential vs. Ag/AgCl in the case of gold over silicon substrate, and −0.6 to 0.7 V applied potential vs. Ag/AgCl when 1D-AAO and 3D–AAO membranes were used as templates (as shown in Figure 1). The assignment of the reduction peaks of Bi^3+^/HTeO_2_^+^ species in solution has been thoroughly investigated by Martin-Gonzalez et al. [15]. If we compare the cyclic voltammetry (CV) obtained with the one obtained when a gold layer over a silicon substrate is used as working electrode (which shows a reduction peak around −35 mV applied potential vs. Ag/AgCl), and with that recorded when a 1D-AAO membrane is used as the working electrode; we can see from Figure 1 an evolution of the reduction peak towards more negative potentials when we go from films (gold/Si substrates) to nanowire network (3D–AAO/gold) to nanowires (1D-AAO/gold substrates). Reaching values > −100 mV in the case of 1D-AAO. This movement of the peak towards more negative values it is believed to be due to the hampered migration of the ions from the solution to the electrode surface when reducing toward narrow nanopores. However, when a 3D–AAO is used as working electrode, the reduction peak appears at an applied voltage of around −60 mV, an intermediate value between that obtained for gold over the silicon substrate and 1D-AAO. Taking into account that both templates, 1D-AAO and 3D–AAO, have the same pore diameter for the longitudinal pores, that is, 40 nm, it has to be the presence of the transversal nanochannels in our 3D–AAO membranes that facilitate the migration of the ions through this kind of alumina matrix. As a consequence, the reduction of the species in dissolution takes place at a less negative potential than the voltage at which the reduction peak of Bi^3+^/HTeO_2_^+^ species appears when using conventional 1D-AAO templates.

**Figure 2:**
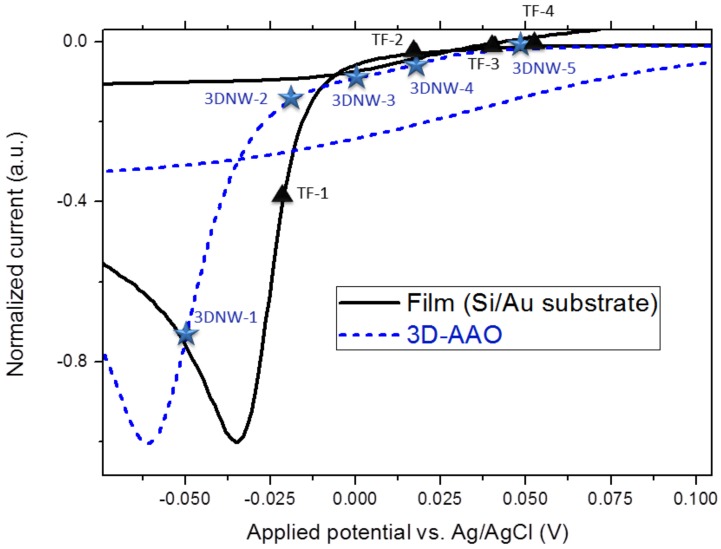
Cyclic voltammetry performed at a scan rate 10 mV/s, showing the different applied voltages at which the nanowire networks (named as 3DNW–#) and films (named as TF–#) were fabricated in pulsed mode.

In order to study the electrochemical route to obtain highly oriented stoichiometric Bi_2_Te_3_ via electrochemical deposition, a thorough study around the reduction peak of the CV (cyclic voltammetry) has to be carried out. In Figure 2, such a study for films (similar to the one in Manzano et al. [13]) and 3D Bi_2_Te_3_ nanowire networks is shown. In both cases, several deposits were performed at different voltages around the reduction peak to determine the optimum potential at which stoichiometric 3D Bi_2_Te_3_ nanowire networks and Bi_2_Te_3_ thin films are grown. 

All the samples (shown in Figure 2 and Table 1) were grown by pulsed electrodeposition alternating between potentiostatic mode, at a constant applied potential (time on) of 1 s, and galvanostatic mode, at zero current density for 0.1 s (time off). The time on/time off ratio was maintained at 10 following the work of reference [13]. Introducing a relaxation time during the electrodeposition process allows a more homogeneous growth front, due to redistribution and recovery of the metal ion concentration at the deposition interface. It also improves the surface morphology of the films [16]. Moreover, it has been reported a dependence between different time offs and the final composition and crystalline orientation of bismuth telluride films [13,17]_ENREF_17 and also in the case of nanowires [18]. Their morphological, structural, and compositional characterization is recorded in Table 1. In the case of films, those grown under an applied potential close to the reduction peak (located at around −35 mV) present a bismuth-rich non-stoichiometric composition, that is, TF–1 (Bi/Te ratio of 2/2.3) and TF–2 (Bi/Te ratio of 2/2.7). However, films grown at less negative potentials and with a difference in the applied voltage with the reduction peak from 75 to 85 mV (TF–3 and TF–4, respectively), present a stoichiometric composition, with Bi/Te ratio of 2/3, along with a preferential orientation along (1 1 0), more marked in the case of TF–4.

In the case of 3D nanowire networks, it was seen that when applying a voltage in the range from 45 to 80 mV higher than the reduction peak (3DNW–2, 3DNW–3 and 3DNW–4) the obtained 3D nanowire network shows the desired stoichiometry, with a Bi/Te ratio of 2/3 and a clear orientation along (1 1 0) direction. For the 3D nanowire networks fabricated applying voltages out of this range, such as 3DNW–1 with a lower voltage difference (10 mV) and 3DNW–5 with a higher voltage difference (108 mV), both showed a non-stoichiometric tellurium-rich composition (Bi/Te ratio 2/3.23 and 2/3.37, respectively).

**Figure 3:**
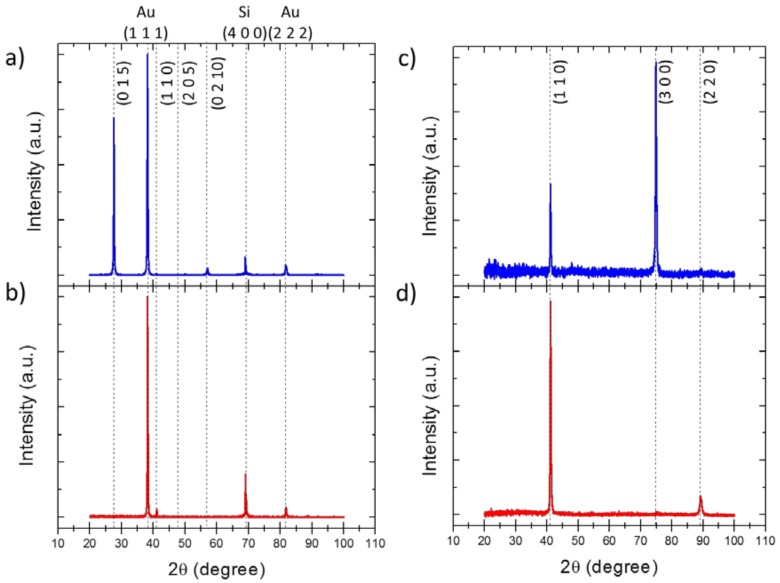
XRD diffraction patterns of films (**a**) TF–1 (non-stoichiometric Bi_2_._31_Te_2.69_) and (**b**) stoichiometric TF–4, and 3D nanowire networks (**c**) non-stoichiometric 3DNW–5 (non-stoichiometric Bi_1_._86_Te_3.14_) and (**d**) stoichiometric 3DNW–4. The blue line has been chosen for non-stoichiometric samples and red for the stoichiometric ones.

A deeper insight into the crystalline structure of both films and 3D nanowire networks can be found in Figure 3. Figure 3a,b show the X-ray diffraction patterns of samples TF–1 and TF–4, respectively. We observe an evolution of the crystalline structure of Bismuth Telluride films when it is grown at potentials near the reduction peak, where they appear oriented along the (0 1 5) for TF–1, to a clear orientation in the (1 1 0) for less negative applied potentials (TF–4). In the case of the non-stoichiometric TF–1 film, the strongest diffraction peak is found at 2θ = 27.60°, which corresponds to the (0 1 5). The second order (0 2 10) and third order (0 3 15) peaks related to the (0 1 5) plane, could also be found at 2*θ* = 57.00° and 2*θ* = 91.62°, respectively. Therefore, in order to determine the preferred orientation of the films, a quantitative analysis was carried out by calculating the Harris texture coefficient, defined by the following expression:

$$T_{C}\left(h\;k\;l\right)=n\;\frac{I_{\left(h\;k\;l\right)}/I_{\left(h\;k\;l\right)}^{0}}{\sum_{1}^{n}I_{\left(h\;k\;l\right)}/I_{\left(h\;k\;l\right)}^{0}},$$

where $I_{\left(h\;k\;l\right)}$ corresponds to the intensity of the (*h k l*) peak observed in the experiment, $I_{\left(h\;k\;l\right)}^{0}$ to the value of this same (*h k l*) peak found in the literature for power diffraction, and *n* is the number of reflections considered in the analysis. Applying this equation, the Harris coefficient obtained for the (0 1 5) orientation was 0.97, that is, highly oriented in this direction. The X-ray diffraction pattern of the stoichiometric film, TF–4, exhibits only two peaks, apart from those of the substrate (Au and Si). A first peak is found at 2*θ* = 41.11°, which corresponds to the (1 1 0). The second peak is located at 2θ = 89.09°, which is related to the second order of the (1 1 0) plane, that is (2 2 0). The absence of any other peaks indicates a preferred crystal orientation along the (1 1 0) plane, with the c-axis oriented in the in-plane direction. This direction in Bi_2_Te_3_ presents a higher thermoelectric performance for out-of-plane applications [6].

In the case of the 3D BiTe nanowire networks, the XRD analysis of the samples (shown in Table 1 and Figure 3c,d) do not show such clear changes of their crystalline structure when the applied voltage is changed, as it was previously seen in the case of the films. Most of the 3D nanowire networks grown present a preferred crystal orientation along the (1 1 0), as it was shown in Table 1. However, non-stoichiometric samples present more peaks in their XRD diffractograms. Figure 3c shows the XRD diffraction pattern of the non-stoichiometric 3DNW–5, where several peaks can be identified at different angles: 2*θ* = 23.57°, 2*θ* = 41.09°, 2*θ* = 74.83° and 2*θ* = 89.29°, which correspond to the (1 0 1), (1 1 0), (3 0 0) and (2 2 0) planes. On the contrary, the XRD diffractogram of the stoichiometric 3D BiTe nanowire networks, such as that shown in Figure 3d for 3DNW–4, exhibit only two diffraction peaks at 2*θ* = 41.11° and 2*θ* = 89.19°, associated to the (1 1 0) and (2 2 0) planes, respectively. Samples 3DNW–2 and 3DNW–3 showed the same XRD pattern. Therefore, we can conclude that stoichiometric 3D Bi_2_Te_3_ nanowire networks grown in this work are strongly oriented along the [1 1 0] direction.

In order to corroborate the EDX results and to study in detail these 3D Bi_2_Te_3_ nanowire networks, Raman shift measurements were performed in all samples. In a recent study by Rodriguez-Fernandez et al. [19], Raman spectroscopy was employed to further investigate the presence of tellurium clusters in Bi_2_Te_3_ nanowires. Taking into account that the non-stoichiometric 3D nanowire networks grown in this work presented tellurium excess, a similar study was carried out in different areas to look for possible tellurium clusters in our structures. The characteristic vibrational modes of bulk single crystal Bi_2_Te_3_, tellurium single crystal, and the samples fabricated in this work can be found in Table 2. 

**Figure 4:**
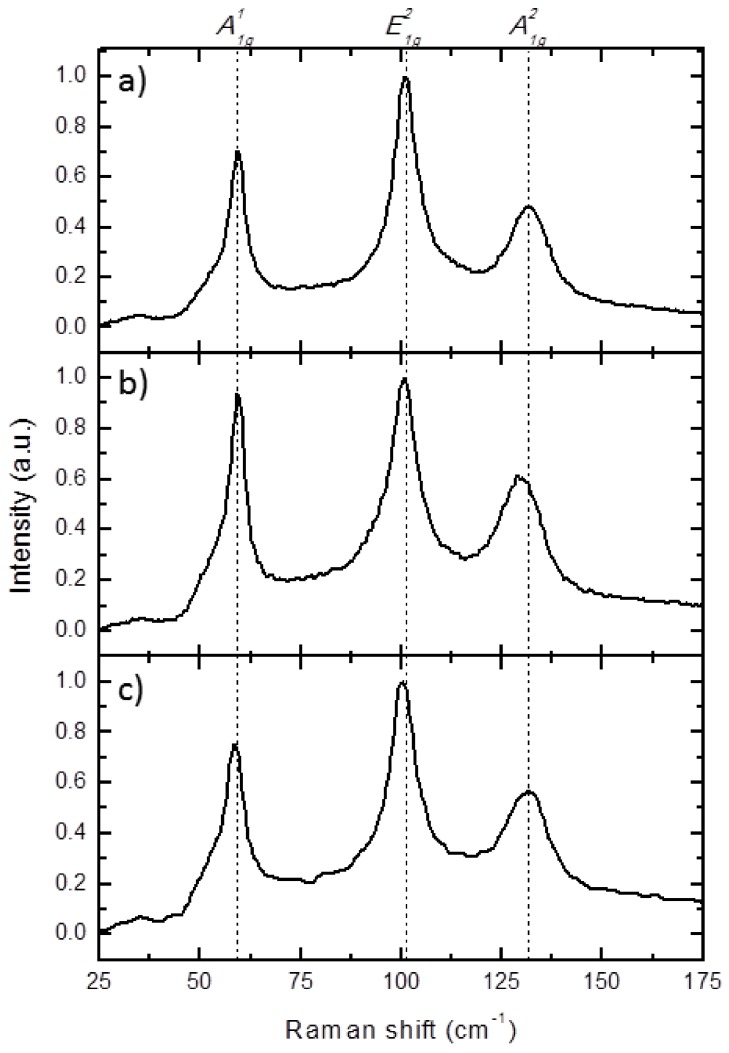
Raman spectra of stoichiometric Bi_2_Te_3_: (**a**) thin film (TF–4), (**b**) conventional 1D nanowire arrays (1DNW–1), and (**c**) 3D nanowires networks (3DNW–4).

As can be seen from the results in Table 2, the peaks found in all cases are associated with the vibrational modes of Bi–Te bonding. In the Bi/Te films measured present a slight shift of the peaks to lower wavelengths when compared to bulk single crystal, as has been previously reported for films [23]. This shift is also present in the case of 1D nanowires (1DNW–1) and 3D nanowire networks. The actual Raman spectra of a stoichiometric Bi_2_Te_3_ thin film (TF–4), 1D nanowire arrays (1DNW–1) and 3D nanowire networks (3DNW–4) are shown in Figure 4a–c, respectively. In all cases, the only peaks we observe are the ones associated with the vibrational modes of Bi_2_Te_3_. No peaks were found around 120 cm^−1^, which would indicate the presence of tellurium clusters in Te-rich Bi_2_Te_3_ nanowires, as it was the case for the nanowires of the work of Rodriguez-Fernandez et al. [19]. The intensities relation between the E_1g_^2^ mode and A_1g_^2^ mode is similar for the three cases, indicating a similar crystallographic orientation [24], as it was already seen by XRD. The main difference is the relative intensity of the A_1g_^1^ breathing mode along the c-axis, which presents in all cases a higher Raman intensity than the higher frequency A_1g_^2^ Raman mode, as it has been observed in the case of studying Quintuplet Layers (QL) of bismuth telluride [25]. The intensity of this A_1g_^2^ mode is similar to thin films and 3DNW, but higher for the 1DNW sample.

**Figure 5:**
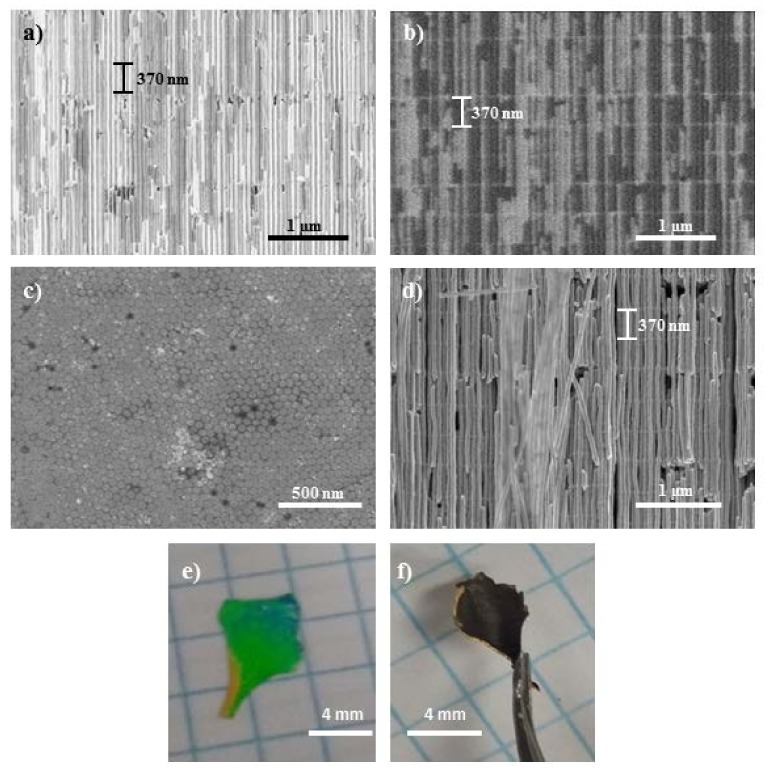
(**a**,**b**) Cross-sectional SEM micrograph images of 3DNW–4 with (**a**) secondary electrons and (**b**) backscattered electrons; (**c**) Top-view SEM image of the 3DNW–4, showing a nanowire diameter of 45–50 nm; (**d**) 3D Bi_2_Te_3_ nanowire network (3DNW–4) SEM micrograph after removing the 3D–AAO template; (**e**) Photograph of a 3D–AAO partially filled with Bi_2_Te_3_ and (**f**) the same sample after chemical etching of the 3D–AAO template, that is, a macroscopic free-standing 3D Bi_2_Te_3_ nanowire network. This sample is free-standing and can be manipulated easily with the help of tweezers.

Finally, Figure 5 shows SEM images of these 3D Bi_2_Te_3_ structures of interconnected longitudinal nanowires, that is, the 3D Bi_2_Te_3_ nanowire network. Cross view images in Figure 5a,b correspond to secondary (a) and backscattered (b) electron images of the sample 3DNW–4, showing the 3D Bi_2_Te_3_ nanowire network grown inside the 3D–AAO template. Backscattering electron images, which are sensitive to the composition, allow a better observation of the transversal channels. Figure 5c shows a top view section of the 3DNW–4. From this image, we can extract the average diameter of the Bi_2_Te_3_ longitudinal nanowires is of 45–50 nm, which is bigger than the diameter of the original 3D–AAO structure, due to the acidic medium in which the electrochemical deposit takes place and which etches the AAO to slightly bigger diameter. Figure 5d shows a cross-sectional micrograph of the 3DNW–4 sample, after etching off the 3D–AAO matrix, that is, a self-standing 3D Bi_2_Te_3_ nanowire network. The distance between transversal channels, chosen to be around 370 nm in this case when fabricating the 3D–AAO template, can be verified from the different cross-sectional images (Figure 5a,b,d), where the interconnections are spaced around 370 nm. Finally, Figure 5e,f show images of the macroscopic structure before (e) and after (f) removal of the 3D–AAO. There it can be seen that the 3D Bi_2_Te_3_ nanowire network can be held with conventional tweezers thanks to the mechanical stability of the connection between nanochannels.

## 4. Conclusions

Here we report a self-standing Bi_2_Te_3_ nanowire network that is interconnected in such a way that the whole structure is mechanically stable so that it can be handled with a pair of tweezers. The fabrication process of these structures consists of a two-step anodization of an aluminum substrate to produce 3D–AAO templates, followed by filling the porous 3D–AAO templates by pulsed electrodeposition. Both techniques allow great control over the structure and growth of these structures, being able to tailor their morphology as well as the composition and crystalline orientation of the material grown inside 3D–AAO membrane. The electrodeposition process has been studied for thin films, 1D nanowire arrays, and 3D nanowire networks in order to obtain stoichiometric samples with Bi_2_Te_3_ composition and the crystalline orientation along [1 1 0] direction, which provides the best thermoelectric efficiency in this material for out-of-plane applications. In the case of the 3D Bi_2_Te_3_ nanowire networks, the optimized composition and crystallographic orientation provide a novel route to obtain a macroscopic nanostructured thermoelectric material based on scalable fabrication processes that could be directly integrated into devices.

## Figures and Tables

**Table 1:** Fabrication parameters, structural and chemical characterization of Bi/Te thin films and 3D Bi/Te nanowire networks fabricated by pulsed deposition.

Sample	Applied Voltage (Difference with the Reduction Peak of the CV) (mV)	Composition	Bi/Te Ratio	Crystalline Orientation	Approx. Growth Rate (nm/min)
3DNW–1	10	Bi_1.91_Te_3.09_	2/3.23	(1 1 0)	33
3DNW–2	45	Bi_1.99_Te_3.01_	2/3	(1 1 0)	32
3DNW–3	60	Bi_1.98_Te_3.02_	2/3	(1 1 0)	27
3DNW–4	76	Bi_2.00_Te_3.00_	2/3	(1 1 0)	16
3DNW–5	108	Bi_1.86_Te_3.14_	2/3.37	(3 0 0)	18
TF–1	33	Bi_2.31_Te_2.69_	2/2.3	(0 1 5)	20
TF–2	50	Bi_2.13_Te_2.87_	2/2.7	(1 0 1)	22
TF–3	75	Bi_1.99_Te_3.01_	2/3	(1 1 0)/(1 0 1)	24
TF–4	85	Bi_2.00_Te_3.00_	2/3	(1 1 0)	15

**Table 2:** Comparison between the vibrational modes of bulk single crystal Bi_2_Te_3_, 3D Bi_2_Te_3_ nanowire networks grown in this work as well as BiTe thin films and Tellurium bulk single crystal and thin films.

Sample	Vibrational Modes (cm^−1^)			References
	**A^1^_1g_**	**E^2^_1g_**	**A^2^_1g_**	
TF–1	59.30	100.70	130.43	This work
TF–4	59.13	100.87	131.91	This work
1DNW–1	59.13	100.87	129.04	This work
3DNW–4	58.42	100.47	131.63	This work
3DNW–5	58.61	99.83	130.61	This work
Bi_2_Te_3_ nanowires	57.80	100.50	127.60	[19]
Bulk single crystal Bi_2_Te_3_	62.5	102	134	[20,21]
	**E**	**A_1_**	**E**	
Tellurium films	91.3	119.7	139.5	[22]
Tellurium thin film	88	117	137	[19]
